# Evaluation of BioFire Respiratory Panel 2 *plus* for Detection of Bordetella pertussis in Nasopharyngeal Swab Specimens from Children with Clinically Suspected Pertussis

**DOI:** 10.1128/spectrum.01806-22

**Published:** 2023-01-05

**Authors:** Chi Li, Chaoying Huang, Ruimu Zhang, Hongmei Wang, Shufeng Tian, Yi-Wei Tang, Jikui Deng

**Affiliations:** a Division of Infectious Diseases, Shenzhen Children's Hospital, Shenzhen, China; b Department of Laboratory Medicine, Memorial Sloan-Kettering Cancer Center, New York, New York, USA; c Department of Internal Medicine, Memorial Sloan-Kettering Cancer Center, New York, New York, USA; Keck School of Medicine of the University of Southern California

**Keywords:** *Bordetella pertussis*, BioFire Respiratory Panel 2 *plus*, respiratory pathogens, children

## Abstract

The objective of this study was to compare the performances of BioFire Respiratory Panel 2 (RP2) *plus*, quantitative real-time PCR (qPCR), and culture for the detection of Bordetella pertussis in nasopharyngeal swab (NPS) specimens. Consecutive NPS specimens were collected from patients with clinically suspected pertussis from 1 March 1 to 31 July 2018 in Shenzhen Children’s Hospital. All the specimens were tested in parallel by RP2 *plus*, qPCR, and culture methods. A total of 464 children were enrolled in this study. The positive pertussis rates of culture, RP2 *plus,* and qPCR were 23.1%, 39.0%, and 38.4%, respectively. Compared to the combined reference standard, the sensitivity, specificity, positive predictive value, and negative predictive values were, respectively, 56.6% (95% confidence interval [CI], 49.2 to 63.7%), 100% (98.3 to 100%), 100% (95.7 to 100%), and 77.0% (72.2 to 81.2%) for culture, 89.9% (84.5 to 93.7%), 96.0% (92.8 to 97.9%), 93.9% (89.1 to 96.8%), and 93.3% (89.5 to 95.8%) for RP2 *plus*, and 86.8% (80.9 to 91.1%), 94.9% (91.4 to 97.1%), 92.1% (86.9 to 95.5%), and 91.3% (87.2 to 94.2%) for qPCR. The most prevalent codetected pathogen was human rhinovirus/enterovirus (*n* = 99, 52.4%), followed by parainfluenza virus (*n* =32, 16.9%) and respiratory syncytial virus (*n* = 29, 15.3%), in children with B. pertussis present, which was consistent with the top three pathogens previously found in children with B. pertussis absent. Turnaround times for RP2 *plus*, qPCR, and culture were 2 h, 8 h, and 120 h, respectively. RP2 *plus* quickly and accurately detected B. pertussis, providing valuable information for an early clinical diagnosis and optimal choice of therapy.

**IMPORTANCE** In recent years, there have been some epidemic or local outbreaks of pertussis in countries with high vaccination rates. One of the crucial factors in controlling pertussis is early diagnosis, which is based on specific laboratory measurements, including culture, serological tests, and PCR assays. Compared to culture and serological tests, PCR is more suitable for clinical application, with a fast detection speed of several hours independent of the disease stage and individual vaccination status. BioFire Respiratory Panel 2 *plus*, a multiplex PCR assay for simultaneously detecting 22 respiratory pathogens, facilitates the quick detection of Bordetella pertussis and coinfecting respiratory pathogens. It also provides valuable information for an early clinical diagnosis and optimal choice of therapy for children with clinically suspected pertussis.

## INTRODUCTION

Bordetella pertussis is a fastidious, Gram-negative, pleomorphic bacillus that causes pertussis, a highly contagious respiratory infection characterized by inspiratory whoop and commonly followed by vomiting that can last for 6 to 10 weeks or longer. Moreover, pertussis may even lead to the death of young children. After the introduction of pertussis vaccines, the morbidity and mortality rates decreased significantly in countries with high vaccination coverage. Nevertheless, since the 1980s, there have been some epidemic or local outbreaks of pertussis in countries with high vaccination rates, termed “pertussis resurgence” (1). In recent years, the incidence of pertussis has also increased in China, consistent with the international trend, but the actual rate of occurrence may still be seriously underestimated (2, 3).

One of the crucial factors leading to “pertussis resurgence” is a change in transmission mode. After mass vaccination, adolescents and adults with low levels of protective antibodies became carriers for transmission of pertussis to infants and young children (4). Due to the atypical symptoms of pertussis in adolescents and adults, specific laboratory measurements, such as culture, serological tests, and PCR assays, now play an essential role in diagnosis (5). However, it remains challenging to isolate B. pertussis from patient specimens, owing to the timing of sampling, the type and quality of specimens, previous antibiotic therapy, and harsh culture conditions for 3 to 7 days. To compare the titer of pertussis toxin-immunoglobulin G (PT-IgG) in serum at the acute stage with that at the recovery stage, at least two specimens from the patient are needed, but they are difficult to obtain in clinical practice, which also hinders its application in early diagnosis. People should not be vaccinated against pertussis within 1 year if diagnosed using only one PT-IgG sample, which is unsuitable for infants and young children (6). In comparison, PCR is more suitable for clinical application, with a fast detection speed of several hours independent of the disease stage and individual vaccination status (7).

The BioFire Respiratory Panel 2 *plus* (RP2 *plus*) is a multiplex PCR assay for simultaneously detecting 22 respiratory pathogens (8). The detected organisms include adenovirus, coronavirus (CoV-229E, -HKU1, -NL63, and -OC43), human metapneumovirus (hMPV), human rhinovirus/enterovirus (HRV/EV), influenza virus A (Flu A, Flu A-H1, FluA-H1-2009, and FluA-H3), influenza virus B (Flu B), parainfluenza virus (PIV-1, -2, -3, and -4), respiratory syncytial virus (RSV), Middle East respiratory syndrome coronavirus (MERS-CoV), B. pertussis, Chlamydia pneumoniae, Mycoplasma pneumoniae, and Bordetella parapertussis. Considering that clinical symptoms alone usually cannot discriminate atypical pertussis from other respiratory infections and that infants with pertussis are often coinfected with other respiratory organisms, RP2 *plus* is more suitable for pertussis diagnosis than a simplex quantitative PCR (qPCR).

Currently, several studies have compared laboratory methods for detecting B. pertussis, including culture, qPCR, BioFire Respiratory Panel (RP), RP2 *plus,* and anti-pertussis toxin IgG serology (8–11). However, data on comparisons between RP2 *plus* and culture methods are limited, particularly in China. In this study, nasopharyngeal swab (NPS) specimens were consecutively collected from children suspected of having pertussis. All the specimens were tested in parallel with RP2 *plus*, qPCR, and culture methods to evaluate the performance of RP2 *plus* for the detection of B. pertussis. The etiology of pathogens in patients with B. pertussis present or absent was also determined.

## RESULTS

### Patients’ demographic and clinical characteristics.

**FIG 1:**
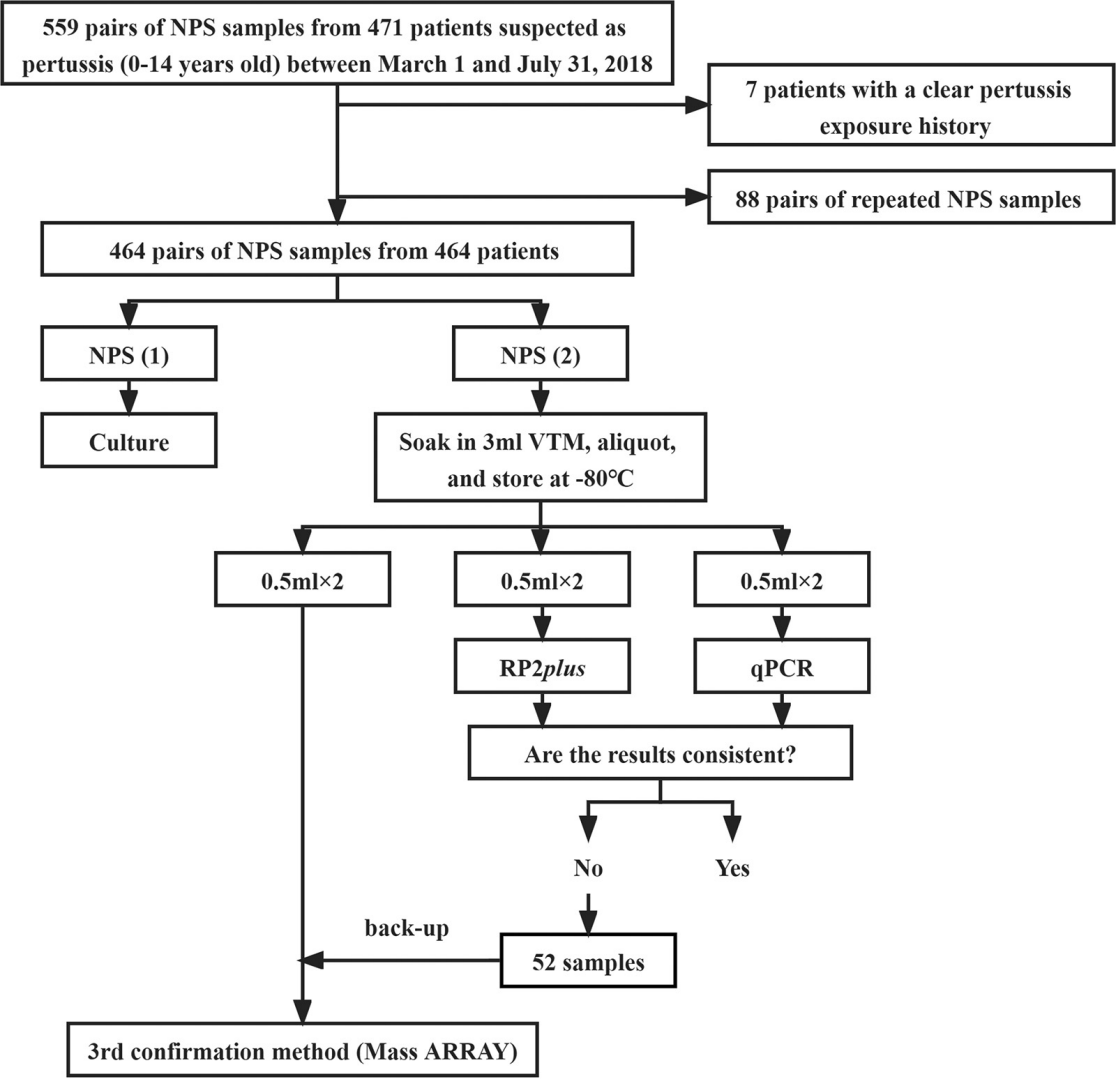
Flow diagram of the study.

**FIG 2:**
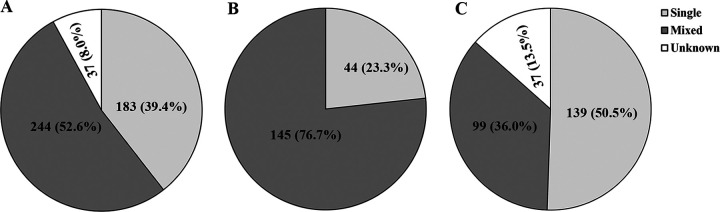
Infection status in all patients (A), patients with confirmed pertussis (B), and patients with probable pertussis (C).

A total of 559 pairs of NPS specimens from 471 patients suspected of having pertussis were initially collected. Subsequently, 7 patients were excluded due to their epidemiologic linkage to a laboratory-confirmed case of pertussis and then 88 duplicate pairs of NPS specimens were removed, which means that only the first pair of samples from each patient was included. Finally, 464 pairs of NPS specimens from 464 patients who satisfied the inclusion criteria and who did not meet the exclusion criteria were enrolled in the study (Fig. 1). Of all patients, 189 children suffered from confirmed pertussis and 275 were probable cases. There were 270 males and 194 females, with a male-to-female ratio of 1.39:1 (Table 1). Overall, 39.4% (183/464) were infected by a single pathogen, 52.6% (244/464) were infected by multiple pathogens, and 8.0% (37/464) had no detectable pathogens (Fig. 2A). Among patients with confirmed pertussis, 44 (23.3%) children were detected with a single pathogen and 145 (76.7%) were detected with multiple pathogens (Fig. 2B), while of the patients diagnosed as probable cases, 139 (50.5%) children were detected with a sole pathogen and 99 (36.0%) were detected with mixed pathogens (Fig. 2C).

**TABLE 1:** Demographics and clinical characteristics of all patients (*n* = 464)

Characteristic	No (%) of patients with:		*P* value
	Confirmed pertussis	Probable pertussis	
Total no.	189	275	
Age at enrollment			
<3 mo	49 (25.9)	47 (17.1)	
≤3–6 mo	71 (37.6)	97 (35.3)	
≤6–18 mo	54 (28.6)	84 (30.5)	
≥18 mo	15 (7.9)	47 (17.1)	0.004
Gender			
Male	106 (56.1)	164 (59.6)	
Female	83 (43.9)	111 (40.4)	
DTaP vaccine (no. of doses)			
0	97 (51.3)	121 (44.0)	
1	28 (14.8)	42 (15.3)	
2	15 (7.9)	27 (9.8)	
3	44 (23.3)	59 (21.5)	
4	5 (2.7)	26 (9.4)	0.004
Cough duration at enrollment			
1–7 days	23 (12.2)	44 (16.0)	
8–14 days	88 (46.6)	52 (18.9)	
15–21 days	45 (23.8)	66 (24.0)	
≥22 days	33 (17.4)	113 (41.1)	<0.001
Hospitalization	131 (69.3)	64 (23.3)	<0.001
Mixed infection	145 (76.7)	99 (36.0)	<0.001

Of children from confirmed cases, 49 (25.9%) were younger than 3 months, 71 (37.6%) were 3 to 6 months old, 54 (28.6%) were 6 to 18 months old, and 15 (7.9%) were ≥18 months old. Of all patients who exhibited possible pertussis, 47 (17.1%) were aged <3 months, 97 (35.3%) were ≤3 to <6 months, 84 (30.5%) were ≤6 to <18 months, and 47 (17.1%) were ≥18 months. The proportion of patients aged ≥18 months with confirmed pertussis was significantly lower than the proportion of those with possible cases (7.9% versus 17.1%, *P = *0.004), which also occurred for the proportion of children who completed booster DTaP (diphtheria, tetanus, and acellular pertussis) vaccination (2.7% versus 9.4%, *P = *0.004) or visited the hospital with a cough duration of ≥22 days (17.4% versus 41.1%, *P < *0.001) (Table 1). In contrast, the rates of hospitalization (69.3% versus 23.3%) and mixed infection (76.7% versus 36.0%) were significantly higher in the group of confirmed cases (*P < *0.001) than in the group with probable pertussis (Table 1).

The health economic data for inpatients demonstrated that patients with confirmed pertussis needed longer hospitalization times and inevitably incurred higher expenses (9.2 ± 4.1 days, RMB 7,479.8 ± 4,306.2) compared to patients with possible pertussis (8.1 ± 4.3 days, RMB 7,274.3 ± 6,053.4) (*P < *0.05). Similarly, expenses were higher for patients who were infected with multiple pathogens (9.1 ± 4.2 days, RMB 7,598.4 ± 4,990.1) than for patients infected with a single pathogen (8.0 ± 4.2 days, RMB 6,626.2 ± 4,544.2) (*P < *0.05). However, no differences were found between patients with B. pertussis infection alone (9.48 ± 4.29 days, RMB 7,348.08 ± 3,550.12) and those with pertussis/virus coinfection (9.13 ± 4.08 days, RMB 7,510.82 ± 4,480.25) (*P > *0.05).

### Results of B. pertussis obtained by culture, RP2 *plus*, and qPCR.

The pertussis detection rates were 23.1% (107/464), 39.0% (181/464), and 38.4% (178/464) for the culture, RP2 *plus*, and qPCR methods, respectively (Table 2). The turnaround time (TAT) included the time of specimen collection and hands-on and instrument time ending with the final result. For RP2 *plus*, qPCR, and culture, TATs were 2 h, 8 h, and 120 h, respectively.

**TABLE 2:** Positivity rates of culture, BioFire RP2 *plus*, and qPCR in all specimens

Assay	No. of specimens		Total no. of specimens	Positivity rate (%)
	Positive	Negative		
Culture	107	357	464	23.1
RP2 *plus*	181	283	464	39.0
qPCR	178	286	464	38.4

Compared to the reference standard, the sensitivity, specificity, positive predictive value (PPV), and negative predictive value (NPV) of culture were 56.6% (95% CI, 49.2 to 63.7%), 100% (98.3 to 100%), 100% (95.7 to 100%), and 77.0% (72.2 to 81.2%), those values for RP2 *plus* were 89.9% (84.5 to 93.7%), 96.0% (92.8 to 97.9%), 93.9% (89.1 to 96.8%), and 93.3% (89.5 to 95.8%), and those values for qPCR were 86.8% (80.9 to 91.1%), 94.9% (91.4 to 97.1%), 92.1% (86.9 to 95.5%), and 91.3% (87.2 to 94.2%), respectively (Table 3). There was no significant difference in sensitivity, specificity, PPV, and NPV (*P* > 0.05) between RP2 *plus* or qPCR and the reference standard in detecting B. pertussis.

**TABLE 3:** Performances of culture, BioFire RP2 *plus*, and qPCR compared to that of the reference^*a*^

Assay and result	Reference standard result (no. of specimens)		Sensitivity (%) (95% CI)	Specificity (%) (95% CI)	PPV (%) (95% CI)	NPV (%) (95% CI)
	+	–				
Culture						
+	107	0	56.6 (49.2–63.7)	100 (98.3–100)	100 (95.7–100)	77.0 (72.2–81.2)
–	82	275				
RP2 *plus*						
+	170	11	89.9 (84.5–93.7)	96.0 (92.8–97.9)	93.9 (89.1–96.8)	93.3 (89.5–95.8)
–	19	264
qPCR						
+	164	14	86.8 (80.9–91.1)	94.9 (91.4–97.1)	92.1 (86.9–95.5)	91.3 (87.2–94.2)
–	25	261	

a NPV, negative predictive value; PPV, positive predictive value; 95% CI, 95% confidence interval.

### Analysis of pertussis detection rates between culture and RP2 *plus*/qPCR in different clinical situations.

When specimens were obtained from patients younger than 18 months, patients with cough durations of ≥8 days at enrollment, or patients with ≤2 doses DTaP vaccine, the detection rates by culture were 23.4% (94/402), 23.2% (92/397), and 21.2% (70/330), respectively. The positivity rates were 42.3% (170/402), 40.3% (160/397), and 42.4% (140/330) with RP2 *plus* and 41.5% (167/402), 39.5% (157/397), and 43.0% (142/330) with qPCR, respectively. The pertussis detection rate observed with culture was significantly lower than the values obtained using RP2 *plus* or qPCR in patients aged <18 months, with a cough duration of 8 days, or with ≤2 doses DTaP vaccine (*P < *0.001).

### Other respiratory pathogens detected by RP2 *plus*.

RP2 *plus* detected at least one pathogen in 427 of the 464 specimens tested, yielding an overall positivity rate of 92.0%. However, no positive detections were observed during the present study for Flu B, Chlamydia pneumoniae, *B. parapertussis*, or MERS-CoV.

**FIG 3:**
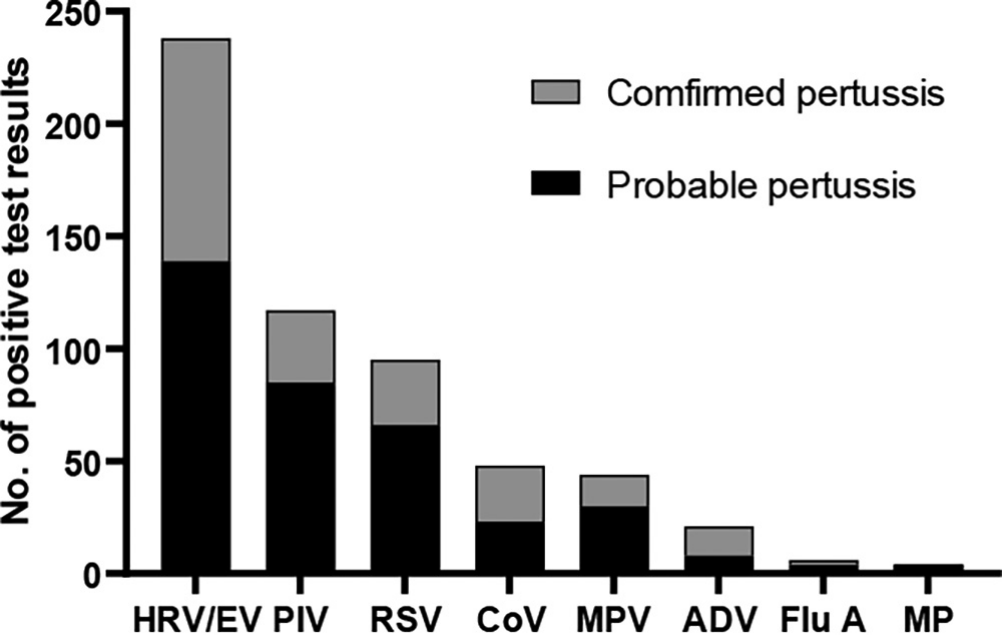
Other pathogens in patients with confirmed and probable pertussis. ADV, adenovirus; CoV, coronavirus; Flu A, influenza virus A; MP, Mycoplasma pneumoniae; MPV, human metapneumovirus; PIV, parainfluenza virus; R/E, rhinovirus/enterovirus; RSV, respiratory syncytial virus.

Coronaviruses were grouped to count detected pathogens, and this was also the case for PIV and Flu A viruses. Thus, apart from B. pertussis, the most prevalent organisms detected during the study were HRV/EV (238 patients, 51.3%), followed by PIV (117, 25.2%), RSV (95, 20.5%), coronavirus (48, 10.3%), hMPV (44, 9.5%), adenovirus (21, 4.5%), Flu A (6, 1.3%), and Mycoplasma pneumoniae (4, 0.9%) (Fig. 3).

### Prevalence of detected pathogens in patients with confirmed pertussis (except for B. pertussis) or probable pertussis.

The top three codetected pathogens in children diagnosed as confirmed cases were HRV/EV (52.4%, 99/189), PIV (16.9%, 32/189), and RSV (15.3%, 29/189). Similarly, HRV/EV, PIV, and RSV were the most prevalent organisms in children with probable cases, which were found in 139 (50.5%), 85 (30.9%), and 66 (24.0%) patients, respectively (Fig. 3).

## DISCUSSION

Traditionally, culture has been considered the “gold standard” pertussis diagnostic test because of its 100% specificity for identification. However, due to the lower sensitivity of culture, we considered the results from molecular biology methods as additional diagnostic information. Previous studies showed that the sensitivity of culture ranged from 12% to 60% (12), while PCR was more sensitive, with estimates ranging from 70% to 99% (13). Likewise, our data indicated that the sensitivities of RP2 *plus* (89.9%) and qPCR (86.8%) were higher than that of culture (56.6%).

The positivity rate of culture was dependent on bacterial activity and the relatively high abundance of the genus Bordetella (14). Furthermore, the timing of specimen collection after the onset of cough, vaccination times, and antibiotic use were closely related to the detection rate of the culture method (9). Similarly, the data showed that when the specimen collection was carried out ≥8 days after coughing onset, the positivity rate of culture was lower than that observed with RP2 *plus* and qPCR, which was also found for patients aged <18 months or with ≤2 doses of DTaP vaccine. In addition, in our study, 327 (99.1%) of 330 patients with ≤2 DTaP doses were aged <18 months, but antibiotic usage history was not collected for these patients. One analysis revealed that 67.4% of 967 Chinese patients with pertussis received antibiotics before hospital admission (15). Therefore, we speculated that many of our patients aged <18 months had been receiving antibiotic therapy before collection of the NPS samples, which likely would harm the detection rates of the culture method.

The genetic target for detection of B. pertussis in RP and RP2 *plus* is *ptxP*, a single-copy gene in the bacterial genome that is B. pertussis specific. The most commonly used B. pertussis target gene in qPCR is IS*481*, a multicopy sequence in several *Bordetella* species (16). Hence, RP2 *plus* or RP has a lower sensitivity but a greater specificity than qPCR, and RP2 *plus* and RP can detect specimens only with a high bacterial load, as previously reported (10, 11). In contrast, some performance studies on RP did not show a lower analytical sensitivity for detecting B. pertussis (17, 18). Our study also revealed no differences in sensitivity or specificity of RP2 *plus* and qPCR in comparison to the reference standard. As to sensitivity, our result may be due to a high abundance of B. pertussis in our patients. Further research is needed to support this hypothesis. Regarding specificity, our result could be linked to the absence of *B. parapertussis* in our patients. This result was inconsistent with a previous study in this region in 2011, which showed that the positivity rates of PCR were 2.0% for asymptomatic *B. parapertussis* infections among schoolchildren (19). Data on the epidemiology of *B. parapertussis* are limited over the world (20). Therefore, monitoring the prevalence of *B. parapertussis* needs to be done in the future.

Mixed infection involving multiple respiratory pathogens is frequently found in infants with coughs (21, 22). For example, coinfections due to B. pertussis in association with respiratory viruses ranged from 20% to 75% (23–25). Likewise, the present study results demonstrated that more than half of the patients (52.6%, 244/464) were infected by multiple pathogens, and among the 189 children with confirmed pertussis, 145 (76.7%) tested positive for respiratory viruses. Additionally, a previous study reported that HRE/EV, PIV, and RSV were the most prevalent codetected pathogens in patients with pertussis and the most frequently detected pathogens in patients with pertussis-like syndrome, characterized by paroxysmal fits of coughing that were not caused by B. pertussis (26). Similarly, the most common pathogens other than B. pertussis detected during our study were HRE/EV, PIV, and RSV.

Theoretically, the economic burden of coinfection is more substantial than that of single infection, with a longer hospital stay and higher hospital costs. And the result of our study also showed that patients infected with multiple pathogens spend about 1 day longer and an extra RMB 1000 than patients infected with a single pathogen. However, the results are mixed about the clinical symptoms and health economic burden for patients with pertussis with or without coinfections (27). Some studies showed that pertussis-RSV coinfections tended to be more severe (26) and were associated with higher rates of wheezing and readmission and a more extended hospital stay (28). Other studies showed that no significant differences were observed in the severity of clinical symptoms in patients with pertussis and those with or without coinfections (29). Furthermore, our data revealed no significant differences regarding the health economic burden between patients with B. pertussis infection alone and those with B. pertussis-virus coinfections. We also looked at coinfections that involved B. pertussis and the top three viruses, and no significant differences in terms of economic burden were found in comparison to B. pertussis monoinfections (data not shown).

Several possible reasons could explain these observations. Children infected with rhinoviruses, enterovirus, PIV, or RSV may be asymptomatic or present with mild to moderate symptoms, which could easily go unnoticed when a patient presents with a pertussis infection (25, 29, 30). Moreover, not all microbes detected by the molecular assays were the “real” causative pathogens. The quantification of the mRNA levels of the pathogen at multiple time points during the entire disease course and/or the measurement of antibody levels may help identify the “real” causative agent. Further studies are needed in this area.

The existence of coinfections involving B. pertussis and other bacteria remains controversial. Most studies have found that other bacteria, particularly Mycoplasma pneumoniae, were not detected in patients with pertussis (22, 31). In contrast, other studies have described patients coinfected with Mycoplasma pneumoniae and B. pertussis (32, 33). Our data found results similar to those of most published studies in that B. pertussis was not detected in the four samples positive for Mycoplasma pneumoniae. Further research is required to identify the cause of the rareness of coinfections due to Mycoplasma pneumoniae and B. pertussis.

Although this was a prospective research study, the time of the samples frozen at −80°C before testing was longer than the 30 days recommended by the FilmArray respiratory panel 2 *Plus* instruction booklet (RFIT-ASY-0136) (34), which may have decreased the positive detective rate of RP2 *plus*. As the study period included only one pertussis peak (March) in the south of China (35), the seasonality information on circulating strains is limited.

### Conclusions.

This study demonstrated the capability of RP2 *plus* in detecting B. pertussis and coinfecting respiratory pathogens with faster TATs, leading to time and labor savings and being more cost-effective for patients.

## MATERIALS AND METHODS

### Study population.

Outpatients and inpatients who had a suspected clinical diagnosis of pertussis entered Shenzhen Children’s Hospital from 1 March to 31 July 2018.

Inclusion criteria included the following: aged 0 to 14 years old; suspected pertussis case by a physician; a cough lasting ≥2 weeks with ≥1 symptoms of paroxysms of coughing, inspiratory “whooping,” and posttussive vomiting; and no epidemiologic linkage to a laboratory-confirmed case of pertussis.

Exclusion criteria included the following: a fever and cough likely to be caused by a severe underlying medical condition, known immunodeficiency, participation in another clinical research study, and preschool pertussis booster vaccination received <1 year before the study began.

Demographic and clinical information, including patient age, gender, pertussis vaccination status, cough duration at enrollment, hospitalization, mixed infection, hospital length of stay, and costs during hospitalization, were collected.

### Specimens.

Paired simultaneous specimens were obtained from each patient with clinically suspected pertussis who entered our hospital from 1 March to 31 July 2018. Some patients may have had duplicate samples collected in the outpatient clinic and the ward. Two Copan flocked swabs (model 516cs01) were used to collect the NPS specimens from each nostril each time. One was used for culture, and the other swab was for molecular biological tests. The molecular testing included RP2 *plus*, qPCR assays, and mass array (Fig. 1).

The swab for culture needed to be plated onto the charcoal agar medium immediately. The plates were incubated for 7 days at 35 to 36°C and inspected on the subsequent third, fifth, and seventh days. If gray, mercury-like colonies appeared, they were subcultured and identified. Plates were discarded as negative if typical colonies did not grow within 7 days.

The swab for molecular tests was soaked in 3 mL of precooled Remel Micro Test M4RT transport medium (VTM) and then divided into six equal parts. Next, all subpackaged samples were kept in the Eppendorf tubes in the refrigerator at 4°C for 1 to 2 h before storage at −80°C for subsequent analysis. Finally, after thawing and vortexing, the inoculated VTMs were tested with RP2 *plus* and qPCR on the same day, with the manufacturer’s instructions followed strictly.

### RP2 *plus* testing.

The RP2 *plus* assay consisted of automated nucleic acid extraction, nested multiplex PCR, and generated endpoint melting curve data analysis. This process took approximately 45 min per run. This multiplex PCR test simultaneously detected B. pertussis by pertussis toxin promoter region (*ptxP*) and 21 other respiratory pathogens, as previously described (8). The result of each target was reported as “detected” or “not detected.”

### qPCR.

DNA extracts were used to identify B. pertussis based on the detection of the sequence 481 gene insertion (IS*481*) (Daan Genetic Co., Ltd., Guangzhou, China), as previously described (36). A threshold cycle value of ≤38 was considered a positive result.

### Discordant analysis.

In the case of discrepant results between RP2 *plus* and qPCR, specimens were retested by a third confirmation method, a molecular mass array. The mass array system used matrix-assisted laser desorption ionization–time of flight mass spectrometry for the precise detection of DNA molecules. IS*481* was used to identify B. pertussis by the third method. Test kit: 10 × PCR buffer (Takara, Japan), 0.25 mM dNTP (Takara), 4 mM MgCl2, 0.5 μM of each amplification primer, PrimeScript^TM^ II reverse transcriptase (Takara), TaKaRa Hot start-Taq polymerase, shrimp alkaline phosphatase (Zhejiang Digena Diagnostic Technology Co., Ltd.), MassARRAY chip (Zhejiang Digena Diagnostic Technology Co., Ltd.). Instrument and manufacturer: ABI 2720 PCR instrument (AB, USA), DP-TOF matrix-assisted laser desorption/ionization-time of flight mass spectrometry (Zhejiang Digena Diagnostic Technology Co., Ltd.), Typer 4.0 software (Zhejiang Digena Diagnostic Technology Co., Ltd.).

### Reference standard of B. pertussis.

A reference standard positive sample was defined as a sample with (i) an organism identified by culture, (ii) a ‘‘consensus positive’’ result, in which an organism was detected by both RP2 *plus* and qPCR, or (iii) an organism detected by two of the molecular assays after discordant analysis.

A reference standard negative sample was defined as a sample with (i) a ‘‘consensus negative’’ result, in which an organism was not detected by RP2 *plus,* qPCR, or culture, or (ii) an organism that was not detected by two of the molecular assays after discordant analysis and for which the culture result was also negative.

A probable or possible case of B. pertussis meets the following clinical diagnostic criteria based on recommendations for the diagnosis and treatment of pertussis in Chinese children: (i) an afebrile condition, (ii) a child aged <4 months with a progressive cough, a child aged 4 months to 10 years with a cough lasting ≥1 week, or a child aged ≥10 years old with a cough lasting ≥2 weeks, and (iii) a child with ≥1 of the symptoms of paroxysms of coughing, inspiratory “whooping,” and posttussive vomiting (6).

A confirmed case of B. pertussis is a probable or possible case with laboratory confirmation, which means that a sample from this case is reference standard positive.

### Statistical analysis.

SPSS v.24.0 (SPSS, Inc., USA) was used for all statistical analyses. Continuous variables are presented as the mean ± standard deviation (SD) if they were normally distributed, and a Mann-Whitney test was employed to evaluate continuous variables. Categorical variables were expressed as frequencies and percentages, and the results were compared using a chi-square or Fisher’s exact test. A *P* value of <0.05 was considered to be a statistically significant finding.

### Ethical approval.

This study was approved by the ethics committee of Shenzhen Children’s Hospital (EC approval number 201703504).
